# Nonlinear absorption and scattering of a single plasmonic nanostructure characterized by *x*-scan technique

**DOI:** 10.3762/bjnano.10.211

**Published:** 2019-11-06

**Authors:** Tushar C Jagadale, Dhanya S Murali, Shi-Wei Chu

**Affiliations:** 1 Department of Physics, National Taiwan University, No.1, Sec. 4, Roosevelt Rd., Taipei 10617, Taiwan; 2 Technical Physics Division, Bhabha Atomic Research Centre, Mumbai 400085, India; 3 Molecular Imaging Centre, National Taiwan University, No.1, Sec. 4, Roosevelt Rd., Taipei 10617, Taiwan

**Keywords:** absorption cross section, laser scanning microscopy, nanoplasmonics, nonlinear absorption, nonlinear scattering, single gold nanostructures

## Abstract

Nonlinear nanoplasmonics is a largely unexplored research area that paves the way for many exciting applications, such as nanolasers, nanoantennas, and nanomodulators. In the field of nonlinear nanoplasmonics, it is highly desirable to characterize the nonlinearity of the optical absorption and scattering of single nanostructures. Currently, the common method to quantify optical nonlinearity is the *z*-scan technique, which yields real and imaginary parts of the permittivity by moving a thin sample with a laser beam. However, *z*-scan typically works with thin films, and thus acquires nonlinear responses from ensembles of nanostructures, not from single ones. In this work, we present an *x*-scan technique that is based on a confocal laser scanning microscope equipped with forward and backward detectors. The two-channel detection offers the simultaneous quantification for the nonlinear behavior of scattering, absorption and total attenuation by a single nanostructure. At low excitation intensities, both scattering and absorption responses are linear, thus confirming the linearity of the detection system. At high excitation intensities, we found that the nonlinear response can be derived directly from the point spread function of the *x*-scan images. Exceptionally large nonlinearities of both scattering and absorption are unraveled simultaneously for the first time. The present study not only provides a novel method for characterizing nonlinearity of a single nanostructure, but also reports surprisingly large plasmonic nonlinearities.

## Introduction

It is well known that the optical properties of plasmonic nanostructures differ significantly from those of the corresponding bulk materials, mainly because of two reasons, i.e., the enhancement in the surface-to-volume ratio and the appearance of resonance effects such as surface plasmon resonance (SPR). For example, the color, or more precisely the scattering and absorption spectra, of metallic nanostructures can be completely different from their bulk counterparts. Plasmonic nanostructures, in general, are characterized by strong scattering, great photo-stability, high brightness and exceptional localization precision. In addition, SPR increases the local electric fields, and thus optical nonlinear interactions are significantly enhanced in metallic nanostructures [1–3]. Nonlinear nanoplasmonics is an emerging field that deals with the nanoscale-confined enhancement of optical fields as well as with the giant nonlinearity provided by plasmonic nanostructures [4–6].

The potential applications of nonlinear nanoplasmonics include nanolasers [7], nanoantennas [8], surface plasmon polariton (SPP)-based waveguides [9], nanostructure-based optical limiters [10], nanoscopy instruments [11–12], and nanoelectronics as integrated optical circuits or transistors for information processing and storage [13]. For the evaluation of plasmonic nonlinear nanophotonics, a technique capable of characterizing the nonlinearity of a single plasmonic nanostructure is highly desirable. Currently, various characterization techniques allow for measurements of nonlinear optical constants such as the absorption coefficient (β) or the refractive index (*n*_2_). These techniques include the *z*-scan method (both β and *n*_2_) [14], degenerate four-wave mixing (only *n*_2_) [15], nearly degenerate three-wave mixing (only *n*_2_) [16], optical Kerr gate and ellipse rotation measurements (both β and *n*_2_) [17], self-phase modulation (only *n*_2_) [18] and Mach–Zehnder interferometry (both β and *n*_2_) [19]. However, please note that all these methods measure nonlinearity in the bulk phase or in thin films [20]. Among them, *z*-scan is probably the most widely adopted technique because of its experimental feasibility and the capability to determine both the nonlinear refractive index and the nonlinear absorption [21]. Below, we briefly address the principle of *z*-scan and its limitations.

The *z*-scan technique is based on measurement of transmittance as a thin sample moves along the propagation path (*z*-axis) of a focused laser beam. The thickness of the sample should be much smaller than the confocal parameter of the beam. Two measurement methods are commonly used, namely open-aperture and closed-aperture *z*-scan. In the open-aperture setup, the transmitted light is completely collected by a large-area power detector. If there is no nonlinearity, the transmittance will be constant no matter where the sample is. However, when there is nonlinear absorption, the transmittance changes as the sample is in the vicinity of the focus, where the intensity is highest along the beam path. Therefore, the open-aperture setup is sensitive to nonlinear absorption and measures the imaginary part of the nonlinear refractive index. In the closed-aperture setup, the transmittance is measured through a small aperture in front of the power detector, so the detected signal is sensitive to beam divergence/convergence, which is determined by the real part of the nonlinear refractive index in the thin sample. When there is no nonlinearity, the transmittance is again constant no matter where the thin sample is. When nonlinear refractive index exists, the sample acts like a *z*-dependent lens that modifies the transmission beam shape. In the closed-aperture method, the power dependency in *z*-direction quantifies the real part of the nonlinear refractive index [21].

In brief, in case of linear responses, the *z*-scan output will be a horizontal line, i.e., constant versus z; while for nonlinear responses, the *z*-scan result deviates from a horizontal line, providing a high-sensitivity detection scheme for nonlinearity. However, *z*-scan measurements typically acquire nonlinear responses from thin samples in which multiple nanostructures are illuminated simultaneously, and collective behavior is monitored. The *z*-scan technique has extensively been applied to study the nonlinear absorption of thin plasmonic films [20], but not that of a single plasmonic nanostructure.

In this study, we propose a different method named *x-scan* to characterize the optical nonlinearity of a single nanostructure. The method is based on laser scanning microscopy, where an excitation beam spot moves in the lateral *x*-direction across a single nanostructure. Similar to the requirements of *z*-scan, but converted into the *x*-direction, the diameter of the nanostructure should be much smaller than the point spread function (PSF) of the laser focus. At low excitation intensities, when there is no nonlinear response, a Gaussian profile of the scanned image due to convolution of the laser PSF and the nanostructure is expected. However, when nonlinearity arises in the nanostructure at higher excitation intensities, the image profile is expected to deviate from the Gaussian profile, thus providing a high-sensitivity detection method for nonlinearity, similar to the *z*-scan technique.

In order to fully characterize the nonlinearity of a single nanostructure, our *x*-scan setup is equipped with two optical detection paths in forward and backward direction, where the former determines the attenuation signal and the latter measures the backscattering signal. Similar to open- and closed-aperture *z*-scan, our *x*-scan technique simultaneously quantifies absorption and scattering, relating to the imaginary and real parts of the refractive index. Applying the novel two-path *x*-scan method to a single gold nanostructure, we have unraveled unprecedented large nonlinearities of both scattering and absorption.

## Results and Discussion

### Microscopic measurement of a single plasmonic nanostructure

**Figure 1:**
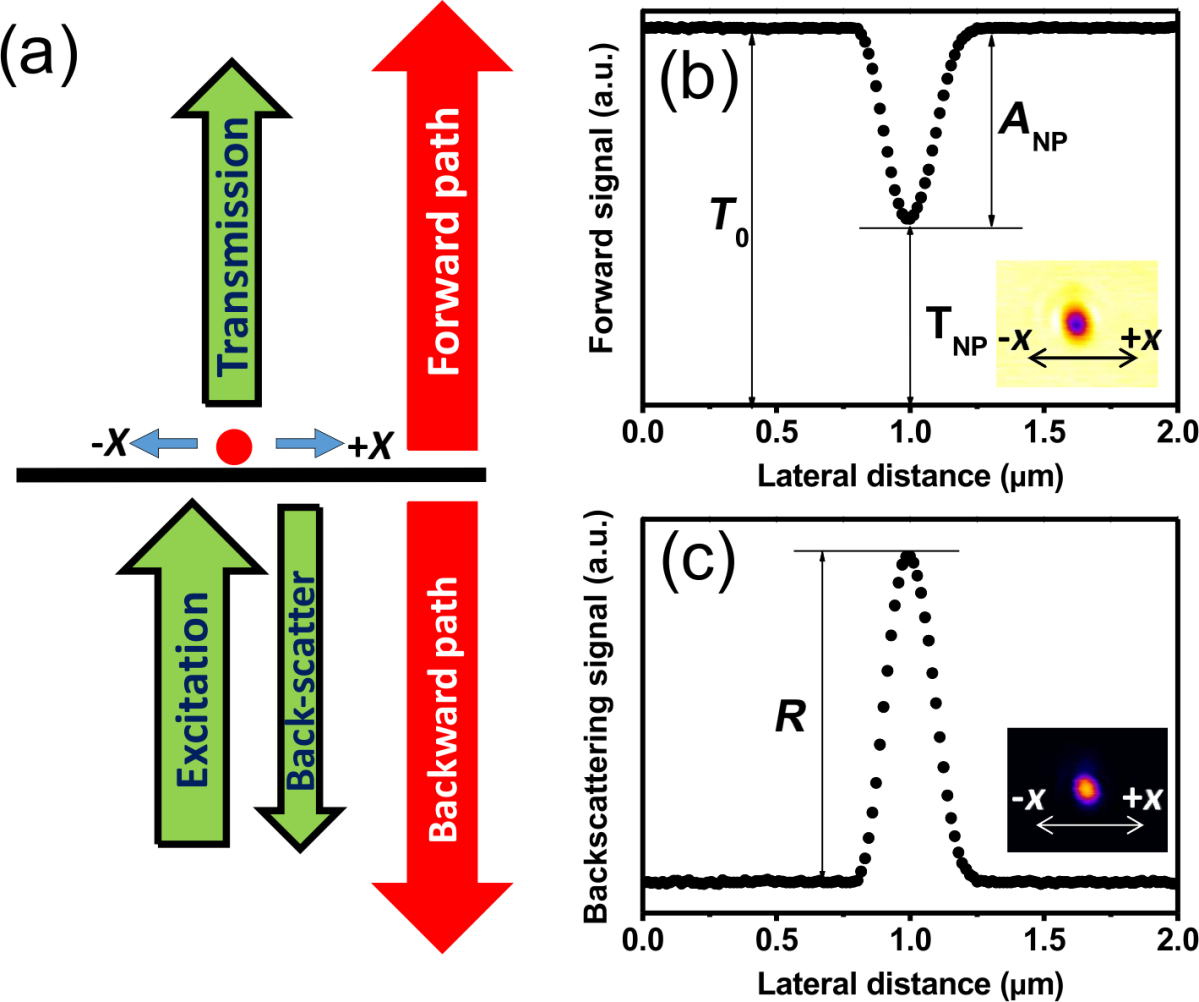
(a) Schematic principle of the *x*-scan method, the excitation focus is scanned in *x*-direction. The back-scattering and transmission of a single nanostructure are separately recorded in backward and forward direction. (b, c) Representative profiles corresponding to the two detection paths. (b) The dip value of the transmission profile gives the attenuation *A*_NP_ of the same single plasmonic nanostructure. *T*_0_ and *T*_NP_ denote the initial laser intensity (no interaction with the nanostructures) and the transmission measured through the gold nanostructure, respectively. The peak value of (c) gives the backscattering intensity *R*.

The idea for characterizing the nonlinear absorption and scattering of a single plasmonic nanostructure using a standard laser scanning microscope is schematically shown in Figure 1a. An inverted microscope is employed with the excitation laser beam in upward direction focused on a single plasmonic nanostructure using an objective with numerical aperture (NA) equal to 1.4. The backscattered light is collected by the same high-NA objective, while the transmitted light is collected by a condenser with an NA of 0.9. Usage of objectives with large NA in both paths ensures efficient collection of the signal of dipole scattering, which is the dominant scattering mode of a small nanostructure [22], in the microscope system. The laser excitation beam is raster scanned in the lateral *x*- and *y*-directions using a pair of galvanometer mirrors, allowing for the observation of the PSF in both the forward and backward detection paths [23].

Figure 1b is a representative linear transmission profile of a single plasmonic nanostructure. The laser-scanning PSF is given as an inset in Figure 1b, having a bright background and a dark spot in the center where the nanostructure is located. The transmitted background represents the total excitation intensity (*T*_o_), which is equal to the sum of the nanoparticle-induced attenuation (*A*_NP_) and the transmission through the nanoparticle (*T*_NP_), i.e., *T*_o_ = *A*_NP_ + *T*_NP_. Note that forward scattering is included in the transmission signal *T*_NP_. Thus, the attenuation signal *A*_NP_ only contains absorption and backscattering. The dark spot in the image, i.e., the Gaussian dip in the *x*-scan signal profile, quantifies the magnitude of attenuation.

Figure 1c is a representative profile of linear backscattering from a single plasmonic nanostructure at low excitation intensity, and the inset gives the laser-scanning PSF. A Gaussian peak is typically observed, and the peak height quantifies the intensity of the backscattering signal (*R*). Importantly, a confocal aperture in the backward detection path provides the capability of optical sectioning, and the nanostructure is typically immersed in oil to remove strong reflection signal from the glass slides (see Experimental section).

From the backscattering and attenuation profiles, the absorption of a single plasmonic nanostructure can be quantified as explained below. It is well known that the total attenuation contains the total absorption and the total scattering (forward and backward). Nevertheless, in our case, *A*_NP_ = *T*_o_ − *T*_NP_, where the transmission through the nanoparticle *T*_NP_ already includes the forward scattering; thus, here the nanoparticle-induced attenuation *A*_NP_ is comprised of the absorption and "only" the backscattering *R* of the nanoparticle. As mentioned above, attenuation and backscattering are monitored in the forward and backward paths, respectively. By checking the linearity of the excitation and detection systems and calibrating the signal intensities with the aid of the glass reflections in the backward and forward paths (Experimental section), the pure absorption signal is obtained by subtracting the backscattering from the attenuation signal, i.e., *A*_NP_ − *R*.

In the following, we report how the attenuation and backscattering signals of a single spherical gold nanostructure with increasing excitation intensity develop from an initial linear Gaussian shape to the nonlinear profiles.

### Linear response: Gaussian PSF

**Figure 2:**
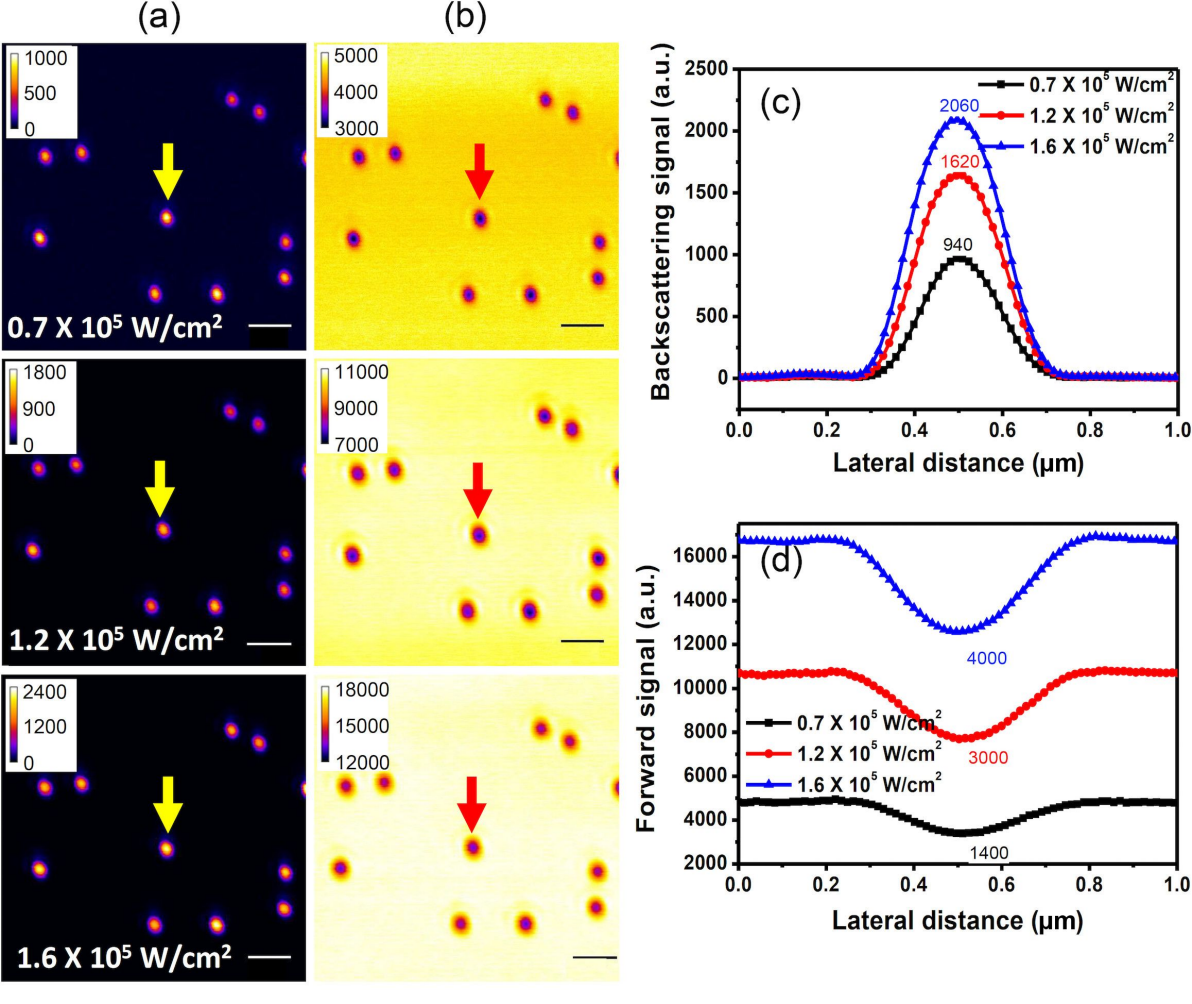
(a) Backward path and (b) forward path images of spherical gold nanostructures in the linear excitation regime. The *x*-scan signal profiles of the spherical gold nanostructure marked by an arrow in (a) and (b) are given in (c) and (d), showing the linear increase of the backscattering and the attenuation signals as functions of the excitation intensity. The number on each peak represents the signal height at the center of the PSF (positive for backscattering and negative for attenuation). The scale bar is 1 µm.

Spherical gold nanostructures dispersed on a glass surface are examined with the two-channel *x*-scan method. Figure 2 shows the power-dependent scattering images and the corresponding signal profiles in the low-power region, manifesting linear responses. Figure 2a and Figure 2b are images acquired in the backward and forward beam paths, respectively. The corresponding excitation intensities are given in each panel. Each image shows ten particles, most of which exhibit similar signal intensities, indicating that the nanoparticles are uniform in size.

Figure 2c and Figure 2d show the respective backscattering and attenuation profiles of the randomly selected single gold nanostructure marked by an arrow in Figure 2a,b. Whether a single nanoparticle has been measured can be examined by inspection of the corresponding scattering spectrum and comparison of the resonance peak with the prediction by Mie theory. The scattering spectrum also helps to monitor changes of the particle size/shape while heating, as we have demonstrated in Figure 2a of [12]. Both the backscattering and the attenuation profiles show a nice Gaussian shape, suggesting that the optical responses are linear at low excitation intensities, as expected. The peak value and dip value (relative to the background) for each curve are given in the figures, showing that the backscattering and attenuation signal intensities indeed increase in proportion with the excitation intensity, further supporting the linear behavior in this excitation intensity range.

### Nonlinear response I: saturation of the PSF

**Figure 3:**
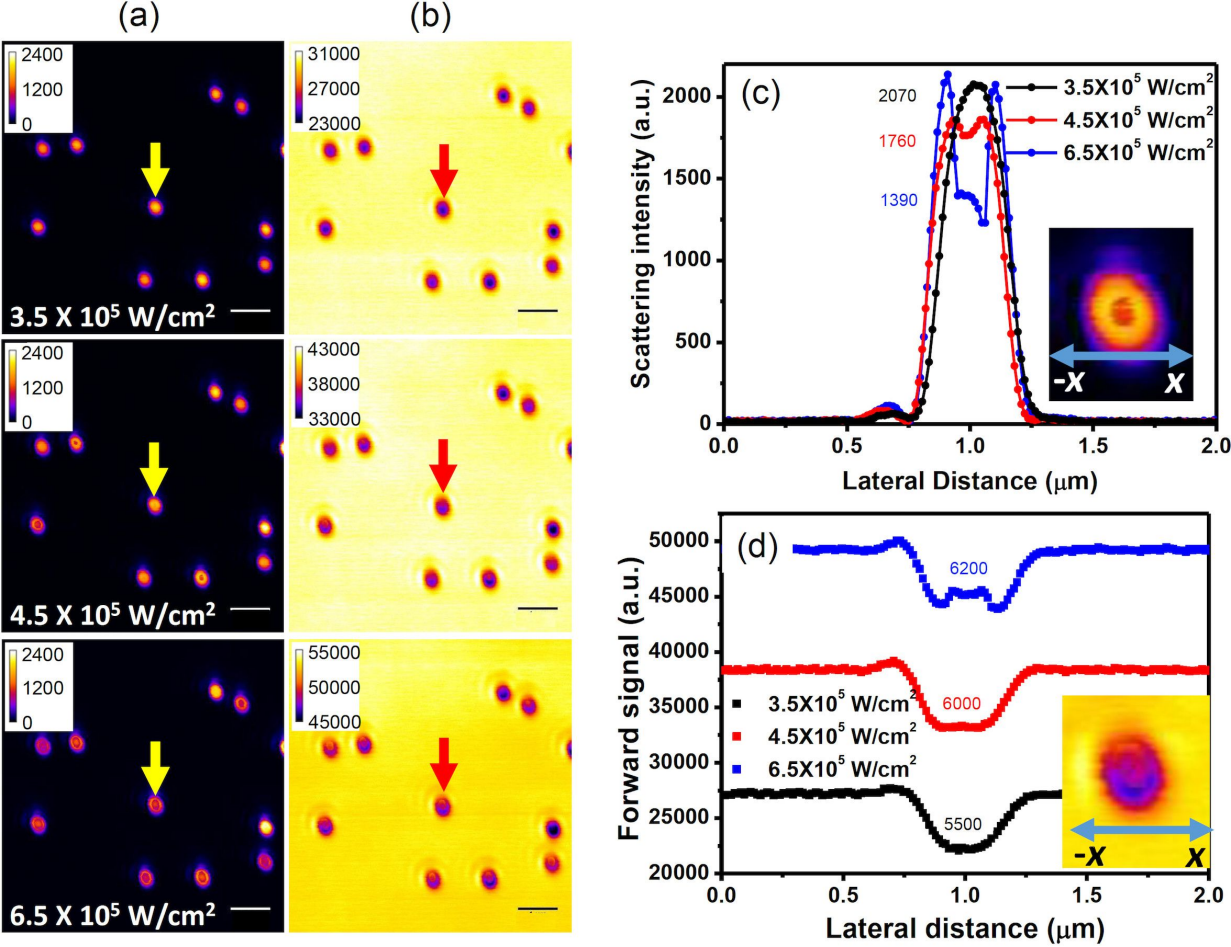
(a) Backward path and (b) forward path images of the same ten spherical gold nanostructures shown in Figure 2 recorded at larger excitation intensities. The *x*-scan signal profiles of the spherical gold nanostructure marked by arrows in (a) and (b) are given in (c) and (d), showing the nonlinear responses of the backscattering and attenuation signals as functions of the excitation intensity. The number on each peak represents the signal height at the center of the PSF (positive for backscattering and negative for attenuation). The scale bar is 1 µm.

When we increase the excitation intensity, interesting changes in the shape of the PSF of the single spherical gold nanostructures are observed. Figure 3a and Figure 3b show the backward path and forward path images recorded at increasing excitation intensities. Figure 3c and Figure 3d give the corresponding scattering and attenuation profiles, respectively. The enlarged images of one randomly selected spherical nanostructure are shown as insets of Figure 3c and Figure 3d. The asymmetry in the enlarged forward path image might be due to the slight misalignment of the condenser in the forward collection path. There are several interesting observations to be made in these figures.

First, both the forward and the backward PSF profiles are no longer of Gaussian shape, indicating the existence of nonlinearities. At an excitation intensity of 4.5 × 10^5^ W·cm^−2^ (red curves in Figure 3c and Figure 3d), nearly flattop PSFs are observed in both channels, manifesting the saturation behavior of both scattering and attenuation.

Second, dips in the intensity peaks are observed in both channels as the excitation intensity increases to 6.5 × 10^5^ W·cm^−2^ (blue curves in Figure 3c and Figure 3d), leading to doughnut-like shapes, as shown in the insets. Since the excitation has a Gaussian profile, the intensity of which is highest in the center of the PSF, the doughnut-shaped responses indicate that the amplitude of the scattering and attenuation decreases with increasing excitation intensity. This is a counterintuitive result, but the doughnut-shaped PSF can indeed be observed for most of the particles shown in Figure 3a and Figure 3b. We will discuss the mechanism of this unexpected nonlinear response later.

Third, as obvious from the numbers on each peak in Figure 3c and Figure 3d, the backscattering signal decreases quickly while the attenuation signal increases slowly. As mentioned earlier, the attenuation intensity contains portions of absorption and backscattering signals, and with a proper calibration, we are able to quantify the percentage of absorption in the two-channel measurement. We derive the absorption as the difference in the scattering and attenuation signals. The results in Figure 3c and 3d indicate that the absorption nonlinearity might be different from the backscattering nonlinearity.

### Nonlinear response II: reverse saturation of the PSF

**Figure 4:**
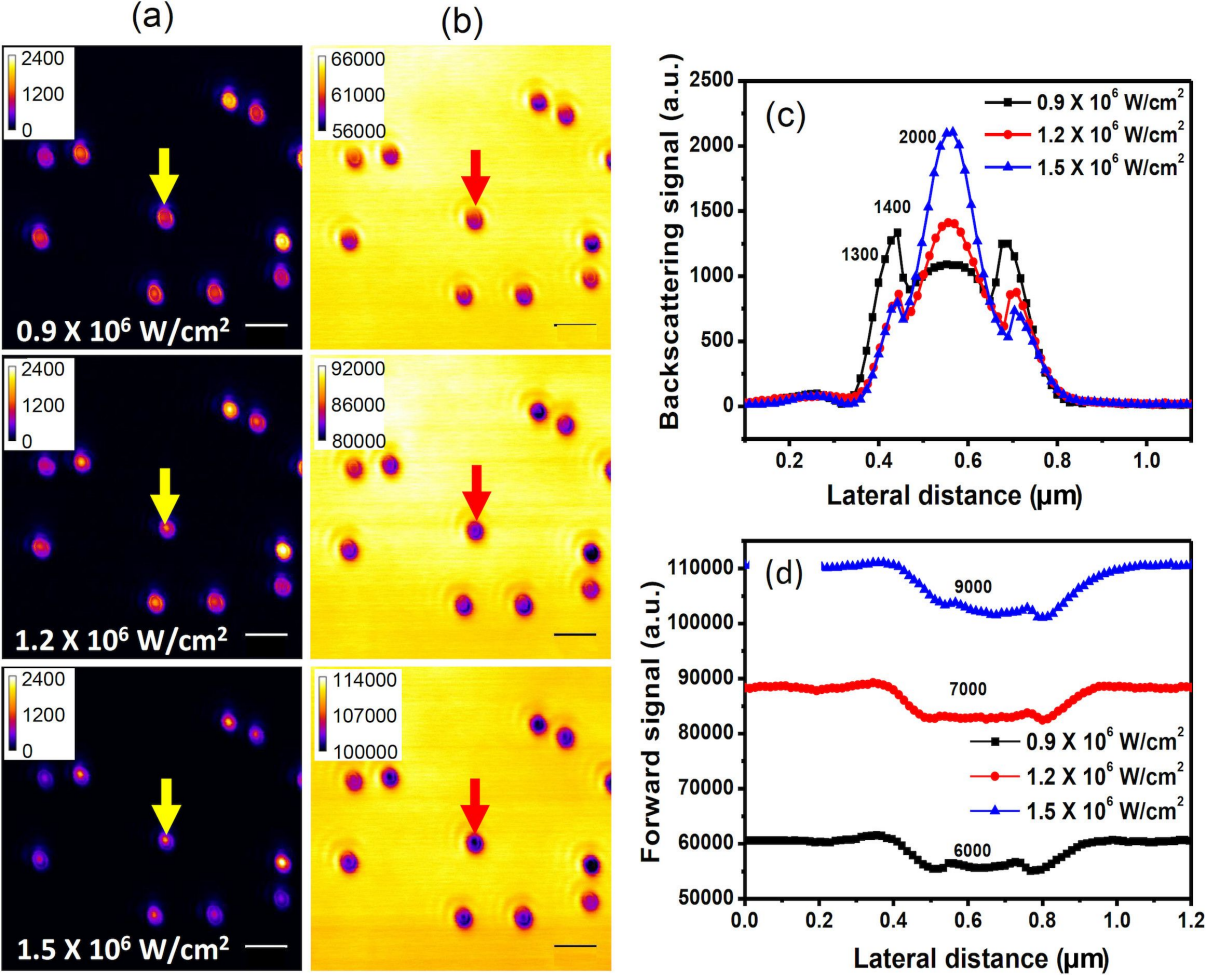
(a) Backward path and (b) forward path images of the same ten spherical gold nanostructures as in Figure 3 at excitation intensities above 10^6^ W·cm^−2^. The *x*-scan signal profiles of the spherical gold nanostructure marked by arrows in (a) and (b) are given in (c) and (d), showing strong nonlinear responses of the backscattering and the attenuation signals versus the excitation intensity. The number on each peak represents the signal quantity at the center of the PSF (positive for backscattering and negative for attenuation). The scale bar is 1 µm.

Upon increasing the excitation intensity above 10^6^ W·cm^−2^, further interesting changes in the PSFs of individual gold nanostructures are observed in both detection paths. Figure 4a and Figure 4b show the backward and forward images of the nanoparticles, and Figure 4c and Figure 4d give the corresponding PSF profiles. Different from the saturation behavior described in the previous section, at excitation intensities of more than ca. 10^6^ W·cm^−2^, a new peak emerges at the center of the PSFs, manifesting a reverse saturation behavior. The phenomenon is more pronounced in the scattering curves depicted in Figure 4c than in the attenuation curves shown in Figure 4d. Yet, the maximum signal values of each of the curves indicate that both scattering and attenuation enter the reverse saturation regime.

**Figure 5:**
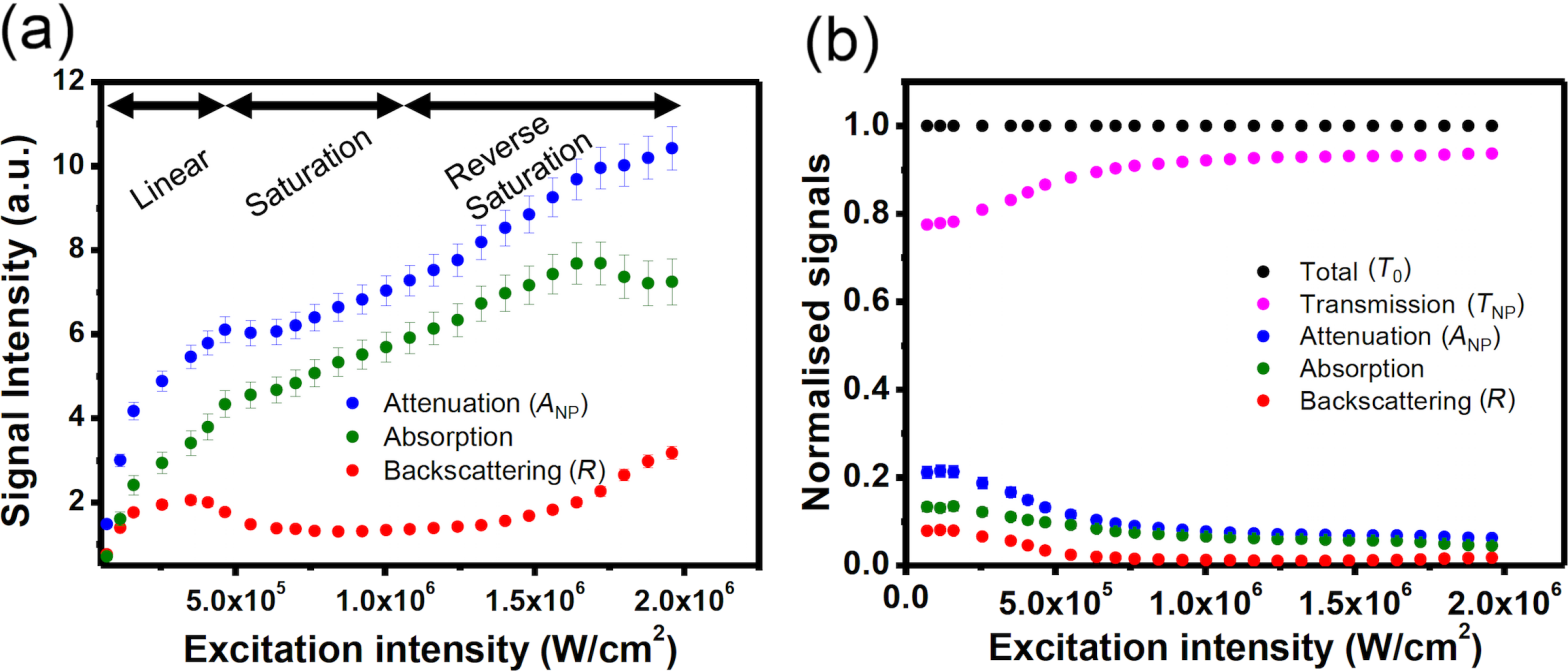
(a) Excitation intensity dependent attenuation (blue dots), absorption (green dots) and backscattering (red dots) signals. (b) Normalized signals (i.e., proportional to the corresponding cross sections) as functions of the excitation laser intensity.

As the excitation intensity increases to 1.5 × 10^6^ W·cm^−2^, a new shape of the peaks emerges, which is most obvious in Figure 4c. Apparently, the new peak shape has a much smaller FWHM compared to the original diffraction-limited PSF (Figure 2). The small FWHM of the reverse saturation peak implies that the amplitude of the scattering and attenuation signals increases faster than the excitation intensity. This means that the power dependency in this region (slope of output versus input) exceeds linearity. Moreover, we did not observe any melting of the nanoparticles in the measurement range, thus, the *x*-scan measurement is fully reversible and repeatable even at the highest excitation intensity of 2 × 10^6^ W·cm^−2^ employed in the measurement (see below in Figure 5). However, once the excitation intensity reaches 5 × 10^6^ W·cm^−2^, the *x*-scan process becomes nonreversible, possibly because the particles are melted or damaged.

### Summarizing the nonlinear behavior

Figures 2–4 depict the backward and forward signals of ten plasmonic gold nanostructures at increasing excitation intensity. Taking the maximum signal intensity values of the corresponding PSF profiles of the backward and forward signals, the excitation intensity dependent attenuation and scattering curves are derived (blue and red dots in Figure 5a). As described in Figure 1, the absorption ratio can be derived as *A*_NP_ – *R* (green dots in Figure 5a). It is very interesting to see that the intensity dependence of the absorption differs strongly from that of the scattering. The latter significantly shows characteristics of saturation and reverse saturation, while the former shows signs of saturation.

Before we compare the nonlinear responses of scattering and absorption in more detail, first, we emphasize that the nonlinearity does not arise from second harmonic generation (SHG) or two-photon luminescence (TPL). Typically, laser intensities of gigawatts per square centimeter are necessary to induce SHG or TPL [24–25]. Yet, in our case, the excitation intensity is on the order of megawatts per square centimeter, hence, three orders of magnitude lower. In our earlier work, we suggested that the physical origin of the observed nonlinearity lies within photothermal plasmonic interactions [12]. Even though the narrow central peak shown in Figure 4c has a large slope when plotted on a double-logarithmic scale, as we have shown in [26], this behavior is different from conventional high-order nonlinearity.

In Figure 5b, we plot the normalized percentage of the different signals (total excitation intensity *T*_0_, transmission *T*_NP_, attenuation *A*_NP_, absorption and backward scattering *R*). The different signals have been defined in Figure 1, and the absorption is given as *A*_NP_ − *R*. Since *T*_0_ increases proportionally to the incident intensity, we normalized it to unity (black dots in Figure 5). All other signals were normalized accordingly. These normalized values can be viewed as the efficiency of the gold nanostructure interacting with light, that should be proportional to the corresponding cross sections. By definition *T*_NP_ (purple dots) + *A*_NP_ (blue dots) = *T*_0_, which is true in the linear and in the nonlinear regime (Figure 5b).

In the linear regime, we find that the backward scattering efficiency is constant at a value of ca. 8% (red dots), which is about half of the efficiency of absorption (14%, green dots). This ratio is consistent with Mie theory, confirming the correctness of our signal calibration. According to Mie theory, the forward scattering ratio should be equal to the backward scattering ratio for this nanostructure. In our measurement, the transmission *T*_NP_ is 79% in the linear region, containing both forward scattering and photons that do not interact with nanostructures. Therefore, the true forward scattering contribution should be only ca. 8%, and about 71% should be due to transmission (no interaction with the nanoparticles). So, the derived ratios of scattering, absorption and transmission agree well with Mie theory.

In the nonlinear regime, i.e., at excitation intensities above 2 × 10^5^ W·cm^−2^, both attenuation and backscattering efficiencies decrease, but interestingly at different rates. Apparently, the particles become more transparent at high excitation intensities, since the attenuation is significantly reduced. The corresponding absorption efficiency also decreases, which means that a saturation of the absorption is observed.

It is remarkable to see that the trends observed for attenuation and absorption in the nonlinear regime are quite different from that observed for backscattering. In the saturation regime (0.2–0.8 MW·cm^−2^), the efficiencies of both absorption and backscattering are reduced by 7% (absorption 13.5% → 6.5%; backscattering 8% → 1%). However, the former changes only by a factor of two, while the latter by a factor of eight. Hence, the backscattering decay is by cubic order larger than the absorption decay.

One possible reason could be that scattering is proportional to the square of the variation of the dielectric constant, while absorption is linearly proportional to the dielectric constant. Within the dipole approximation, the absorption and scattering cross sections of a plasmonic nanosphere can be determined by classical Mie theory as:

[1] cabs=4πkr3Im(εp−εmεp+2εm)

[2] $$c_{\text{sca}}=8\pi k^{4}r^{6}{\left|\frac{\varepsilon_{\text{p}}-\varepsilon_{\text{m}}}{\varepsilon_{\text{p}}+2\varepsilon_{\text{m}}}\right|}^{2}$$

where, *k* is the wave vector, *r* is the radius of the particle, ε_p_ is the dielectric constant of the particle and ε_m_ is the dielectric constant of the surrounding medium. Upon irradiation with high-intensity laser light, the photothermal effect induces a change of the particle permittivity leading to the nonlinearity. However, this equation only explains a square-order difference between scattering and absorption.

The above equation considers the total scattering cross section. Nevertheless, in our experiment, only backscattering is monitored. Therefore, another possible factor is asymmetric scattering due to the interference of high-order multipoles, which means that the efficiency of backscattering is no longer similar to that of forward scattering. Recently, there were many reports [27–29] on directional scattering effects due to multipole interferences in plasmonic nanostructures. However, most of them refer to specially designed structures for which the magnetic and the electric dipoles couple. More studies are necessary to prove the possibility of directional scattering in heated plasmonic nanostructures by simultaneously recording the efficiency of forward and backward scattering.

In addition to the interference of multipoles, in the forward direction, the light scattered in forward direction could also interfere with the transmitted light, such that the angular distribution might change. However, the forward scattered light is collected using a condenser of NA 0.9, hence, the collection angle is ±64°. Thus, most of the scattered light should be registered regardless of whether such interference occurs or not. Moreover, no interference patterns were observed in the forward image, and we have confirmed in the linear regime that the derived ratios of scattering, absorption and transmission agree well with Mie theory. Therefore, even if interference may occur in the forward direction, it does not influence the absorption efficiency in our case.

Another difference in the backscattering and absorption efficiencies shown in Figure 5 is that only the former exhibits a clear reverse saturation effect. This might be due to the additional thermal effect of the surrounding medium (immersion oil). Once again, further studies will be necessary to investigate the temperatures of the nanostructure and of the immersion medium, to provide a better explanation for the complicated photothermal nonlinearity.

## Conclusion

In this report, we successfully demonstrate the simultaneous measurement of nonlinear attenuation, absorption and scattering in a single plasmonic nanostructure, for the first time using the two-path *x*-scan method. In contrast to the *z*-scan technique, the *x*-scan method, which is based on a laser scanning microscope, is capable to characterize and visualize the nonlinear responses from the PSF of a single nanostructure. With the simultaneous measurement of forward and backward scattering, we could quantify the nonlinearity of absorption and scattering, which show surprisingly different behaviors. This may lead to the possibility of directional emission by a single heated nanostructure.

## Experimental

**Figure 6:**
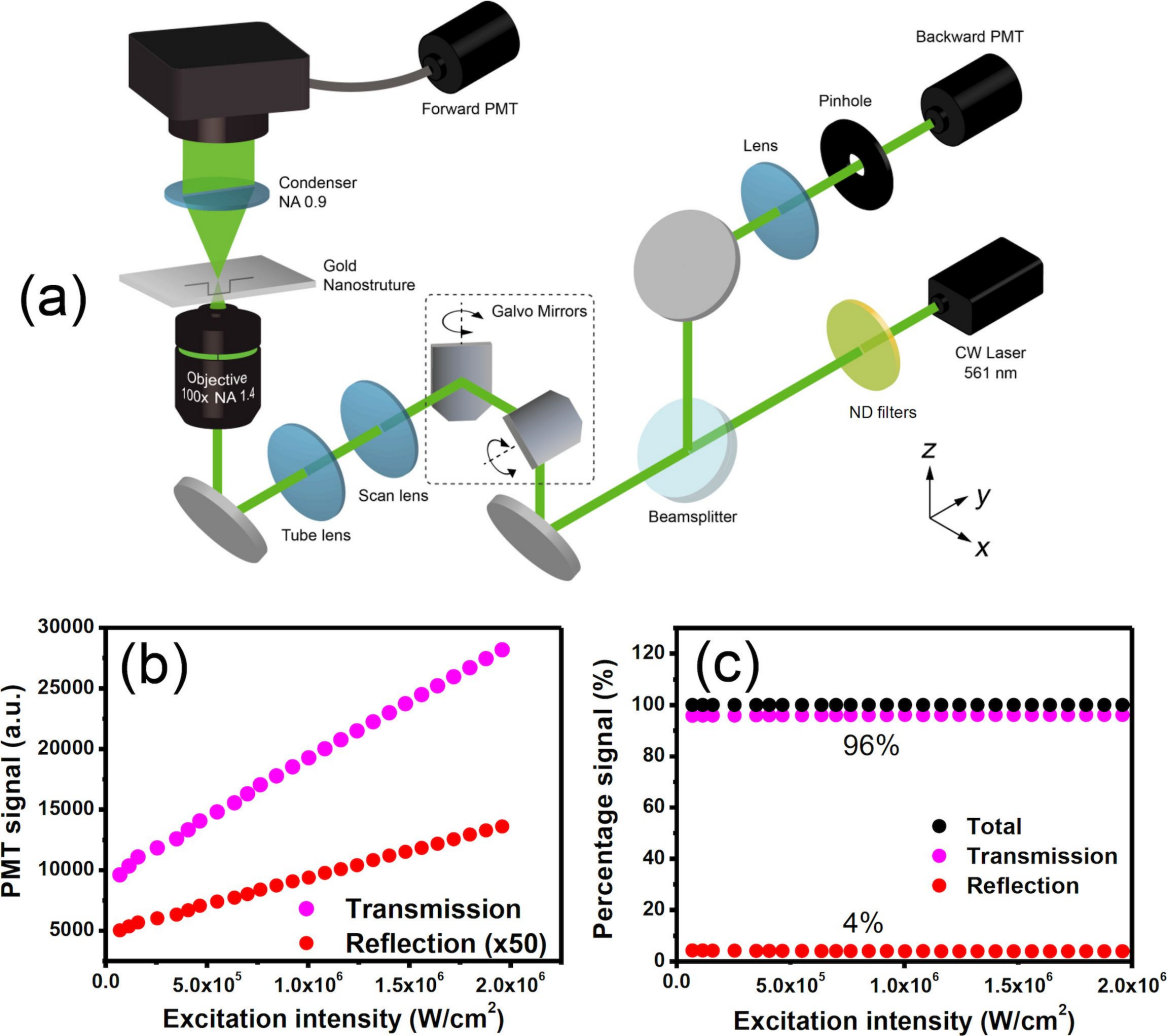
(a) Schematic of the experimental setup of the laser scanning system based on an inverted microscope. (b) Linearity test of the backward and forward signals from the partial reflection and transmission of a cover glass. It is obvious that no nonlinearity is induced by the microscope system. (c) The forward and backward signals are calibrated to correctly represent 4% reflection and 96% transmission from an air–glass interface. The same calibration applies to all signal processing in the main text.

The experimental setup, which is based on a modified confocal laser scanning microscope (IX71+FV300, Olympus, Japan) is shown in Figure 6a. A CW laser beam of wavelength 561 nm (Jive™ 561 nm, Cobolt, Sweden) was sent through a pair of built-in galvanometer (galvo) mirrors and then focused on plasmonic nanostructures by an objective (UPlanSApo 100x/NA1.4, Olympus, Japan) to form a two-dimensional raster scanning at its focal plane. The power of the excitation beam was fine-tuned through neutral density (ND) filters. The backscattered signals of the plasmonic nanostructures were collected through the same objective, separated from the incident beam with a 50/50 beam splitter, spatially filtered by a confocal aperture and finally detected by a backward photomultiplier tube (PMT) detector. On the other hand, the transmission signal was collected by a condenser (U-LTD/NA 0.9, Olympus, Japan) and was monitored by the forward PMT detector directly without confocal aperture. Both forward path and backward path images were formed on a computer by synchronizing the PMT signals and the scanner. Due to the different collection paths and PMT sensitivities in forward and backward directions, it is important to calibrate the signals in order to determine the absorption signal, as shown below.

Figure 6b shows the forward (transmission) and the backward (reflection) signals of a cover glass when gradually increasing the excitation intensity. Here, the backward signals came from the air–glass interface reflection at the top of the cover glass, while the transmitted photons make up the forward signal. The perfect linear dependency of both signals verifies that no nonlinearity is induced by the optical excitation and detection system. In addition, the air–glass reflection should be 4% and the transmittance 96%. This way, we made sure that the forward and backward signal were calibrated accurately (Figure 6c). The same calibration scheme was applied to yield Figures 2–5 in the main text, and to derive the ratio of scattering, absorption and transmission. Since the results agree well with the prediction of Mie theory, the reflectivity estimation should be reasonable.

### Sample preparation

As samples, we used 80 nm diameter gold nanospheres commercially available from BBI Solutions, UK. Before use, the nanostructure solution was sonicated for 2 min to avoid particle aggregation. Then, one drop of the solution was placed on polysine slides (Thermo Fisher Scientifics, MA) for 20 s, which was subsequently gently rinsed with deionized water and dried in a nitrogen stream. The sample was immersed in index-matching oil to remove reflections by the glass.
